# From Melt Processing to Composting: Solvent-Free Thermoplastic Starch/Polyvinyl Alcohol Blends with Tailored Structure and Performance

**DOI:** 10.3390/polym18111333

**Published:** 2026-05-28

**Authors:** Franciszek Pawlak, Cristina Pavon, Harrison de la Rosa-Ramírez, Miguel Aldas, Dana Luca Motoc

**Affiliations:** 1 Instituto Universitario de Tecnología de Materiales, Universitat Politècnica de València, 03801 Alcoy, Alicante, Spain; magwisz@gmail.com (F.P.); hardela@epsa.upv.es (H.d.l.R.-R.); 2 Centro de Investigaciones Aplicadas a Polímeros, Departamento de Ciencia de Alimentos y Biotecnología, Facultad de Ingeniería Química y Agroindustria, Escuela Politécnica Nacional, Quito 170517, Ecuador; miguel.aldas@epn.edu.ec; 3 Department of Automotive and Transport Engineering, Transilvania University of Brasov (UniTBv), 1 Politehnicii Street, 500024 Brasov, Romania; danaluca@unitbv.ro

**Keywords:** thermoplastic starch (TPS), polyvinyl alcohol (PVA), solvent-free extrusion, injection molding, composting disintegration

## Abstract

Thermoplastic starch (TPS) and polyvinyl alcohol (PVA) blends were prepared over the full compositional range (0–100 wt% PVA) by solvent-free twin-screw extrusion and injection molding. This enabled a systematic evaluation of structure–performance–disintegration relationships under industrially relevant conditions. The blends exhibited clear composition-dependent trends in thermal and mechanical behavior. Increasing PVA content from 0 to 100 wt% raised the onset degradation temperature (T5%) from 160.5 °C to 290.5 °C and increased crystallinity from near zero to 12.24%. Mechanically, the response evolved from a rigid TPS-rich state to a ductile PVA-rich one. FTIR and SEM analyses indicated partial compatibility, with limited molecular-level interactions leading to morphologically homogeneous but only partially miscible blends. Under simulated composting conditions, all formulations showed substantial physical disintegration. PVA-rich blends (≥60 wt%) disintegrated rapidly (>80% mass loss within two days), primarily by dissolution rather than microbial degradation. Overall, this work provides a comprehensive, scalable assessment of solvent-free TPS/PVA blends, clarifies their limited compatibility under melt processing, and demonstrates how composition can be used to tailor structure, performance, and disintegration behavior across the full compositional range.

## 1. Introduction

Growing environmental concerns over plastic waste have intensified the search for sustainable alternatives, leading to increased interest in bioplastics [1]. Global bio-based plastics production capacity currently represents roughly 0.5% of the 431 million tonnes of plastics produced annually, yet the sector is expanding rapidly, with production capacities projected to grow from 2.47 million tonnes in 2024 to approximately 4.69 million tonnes by 2030. Among bio-based materials, starch-based plastics are particularly prominent due to their natural abundance, low cost, biodegradability, and biocompatibility, accounting for approximately 350,000 tonnes of annual production in recent years and sustaining a compound annual growth rate of around 12% [2,3]. However, native starch lacks thermoplasticity and requires destructuring and plasticization to form thermoplastic starch (TPS) [4].

Despite its advantages, the widespread application of TPS is hampered by inherent limitations, including poor mechanical properties and high moisture sensitivity [5]. A common strategy to overcome these drawbacks is to blend TPS with other biodegradable polymers that can act as a reinforcing phase. Among potential blend partners, polyvinyl alcohol (PVA) is particularly promising. PVA is a synthetic, water-soluble polymer known for its excellent film-forming capacity, high mechanical strength, remarkable oxygen barrier properties, and full biodegradability [6,7,8,9]. These properties make it valuable in applications ranging from barrier films and packaging to hydrogels [10,11,12].

Due to its complementary characteristics, PVA is often blended with natural polymers like starch to create materials with balanced performance and cost [13]. Hydrogen bonding between the abundant hydroxyl groups of TPS and PVA is often proposed as the main mechanism promoting compatibility [14]. In this context, however, the role of glycerol—used as a TPS plasticizer—deserves attention, as it may partially migrate into the PVA phase during melt processing due to the thermodynamic affinity between glycerol’s hydroxyl groups and PVA’s hydroxyl-rich backbone. Such plasticizer redistribution has been reported in starch-based blends and may influence the glass transition temperature, crystallinity, and mechanical response of the PVA phase, adding complexity to the interpretation of composition-dependent properties [15,16].

Consequently, TPS/PVA blends have been widely studied. However, a critical analysis of the literature reveals a significant methodological gap. The vast majority of these studies rely on the solvent casting method for blend preparation [17,18,19,20]. Although effective, solvent casting has several drawbacks: poor scalability, high energy demand, and artificial enhancement of polymer compatibility due to solvent-mediated interactions. Furthermore, challenges in dispersing or dissolving high concentrations of either component often restrict these studies to blends where one polymer is a minor constituent, typically below 10–30% [21,22]. For instance, Rabe et al. and Ai et al. focused on PVA concentrations around 10% as a reinforcing agent for TPS [21,23]. Studies attempting to overcome solubility issues, for example by using ionic liquids as processing aids, introduce a third component that complicates the interpretation of the fundamental TPS-PVA interaction [24]. As a result, the true compatibility, processability, and property evolution of neat TPS and PVA across the entire compositional spectrum, under industrially relevant, solvent-free conditions, remain inadequately explored.

In this work, TPS/PVA blends were produced by twin-screw extrusion and injection molding to replicate industrial conditions and overcome the limitations of solvent casting. During blending, the absence of supplementary liquids allowed exclusive examination of the interaction between the two polymers. This approach provides a practical alternative to solvent casting, enabling melt blending across the full TPS/PVA compositional range under industrially relevant conditions and yielding uniform specimens free of voids or bubbles. Most previous TPS/PVA studies have focused on compositions with ≤10 wt% of one component, because dispersing or dissolving higher fractions in solvent-cast systems is challenging. In contrast, the injection-molding route employed here permits investigation of the entire 0–100 wt% range, allowing the mutual compatibility and processing stability of TPS and PVA to be examined under solvent-free conditions. Beyond confirming known TPS/PVA interactions, this study systematically evaluates structure, thermal–mechanical behavior, and disintegration across the full compositional range and establishes composition–processing–performance relationships for solvent-free TPS/PVA blends under scalable, industrially relevant conditions.

## 2. Materials and Methods

### 2.1. Materials

This study used thermoplastic starch (TPS) and polyvinyl alcohol (PVA). TPS was obtained by processing food-grade corn starch containing 27% amylose, provided by Cargill^®^ (Martorell, Barcelona, Spain), and glycerol, with a purity of 99%, provided by Panreac^®^ (Barcelona, Spain). Polyvinyl alcohol (PVA) Mowiflex C17 was supplied by Kuraray Europe GmbH (Hattersheim am Main, Germany), with a glass transition temperature of 59.4 °C and a melting temperature of 176.6 °C. The C17 commercial grade exhibits a suitable processing temperature window.

### 2.2. Polymer Blends Manufacturing

#### 2.2.1. Stage 1: TPS Production

To obtain the TPS native starch and PVA were dried at 60 °C for 24 h prior to processing. Then, the native corn starch and glycerol were mixed in 75 wt.% and 25 wt.%, respectively, to obtain a homogenous mixture and starch plasticizer absorption. The materials were mixed and hermetically sealed in a container. Then, the materials were extruded in a Construcciones Mecanicas Dupra SL (Castalla, Spain) co-rotating twin-screw extruder with an L/D ratio of 25 and a temperature profile of 130-135-140-150 °C (from hopper to die) and with a rotation speed of 45 rpm. This temperature profile promotes efficient glycerol absorption and prevents starch retrogradation, which typically occurs above 200 °C. TPS was ground in a Silmisa mill (Onil, Spain) to obtain pellets between 3.5 and 4 mm with an L/D ratio between 1 to 1.5. In this work, TPS was used as a single-component material in which glycerol acts as an integral plasticizer; consequently, the TPS/PVA system is treated as a binary blend throughout.

#### 2.2.2. Stage 2: TPS/PVA Blend Processing

The pre-produced TPS pellets were subsequently blended with PVA in a second extrusion step. The TPS/PVA blend formulations and their codes are summarized in Table 1. All TPS/PVA blends were processed under identical parameters. The blends were extruded in the Dupra SL extruder, using a temperature profile of 165-175-185-185 °C (hopper to die), with a rotation speed of 40 rpm.

This higher temperature range was necessary to ensure complete melting and homogeneous mixing of the PVA phase (T_m_ ≈176.6 °C). Since starch had already been converted to TPS in Stage 1, the glycerol-plasticized matrix remains thermally stable at these temperatures, well below the onset of TPS thermal degradation (~200 °C). The materials were milled with Silmisa mill and injected-molded to obtain standard test samples using a Sprinter-11, injection molding machine (Erinca S, L, Barcelona, Spain) using a processing temperature profile of 165-175-185 °C (from hopper to die, with an injection speed of 65%, and a screw speed of 45%, a back pressure of 8%, holding pressure 20%, cushion of 10, holding time of 3.5 s, and cooling time of 25 s. The machine displays settings as percentages rather than absolute units.)

### 2.3. Thermal Characterization

Thermogravimetric analysis (TGA) was conducted using a TGA1000 thermogravimetric analyzer (Linseis, Germany) under nitrogen (20 cm^3^ min^−1^) at 10 °C min^−1^ from 35 to 700 °C. The onset degradation temperatures (T_5%_) were determined at 5% of mass loss. The temperature at the maximum decomposition rate (T_max_) was obtained from the derivative (DTG) curves.

Differential scanning calorimetry (DSC) was performed using a DSC821e scanning calorimeter (Mettler Toledo, Switzerland) with a heating–cooling–heating cycle from 30 to 300 °C at 10 °C min^−1^ under nitrogen (20 cm^3^ min^−1^). Thermal properties were evaluated based on the DSC curves first cooling and second heating. The PVA’s glass transition and melting temperatures (T_g_ and T_m_), as well as the melting and crystallization temperatures of TPS (T_m_ and T_c_) were determined directly from the differential scanning calorimetry (DSC) curves. The degree of crystallinity ($X_{c}$) was calculated using Equation (1):

(1) $$X_{c}\%=\frac{{\Delta H}_{m}-{\Delta H}_{\mathrm{CC}}}{f\;\times \;{\Delta H}_{m}^{c}}\times 100,$$

where $\Delta H_{m}$ is the measured melting enthalpy, $\Delta H_{\mathrm{cc}}$ is the cold crystallization enthalpy of PVA, ${\Delta H}_{m}^{c}$ = 138.6 J g^−1^ (for fully crystalline PVA [25], and f is the PVA weight fraction.

### 2.4. Mechanical Characterization

Mechanical characterization of the blends was carried out by tensile, flexural, hardness, and impact tests. The tensile test followed ISO 527-1:2019 [26] on five specimens per formulation, and the flexural test followed ISO 178:2019 [27] on five specimens per formulation. Both tests were performed on an ELIB-50-W universal testing machine (Ibertest, Madrid, Spain) equipped with a 5 kN load cell and a crosshead speed of 10 mm/min. Impact resistance was measured on a Charpy pendulum impact tester (Metrotec, San Sebastián, Spain) with a 1 J pendulum, using five unnotched specimens per formulation in accordance with ISO 179-1:2025 [28]. Indentation hardness was evaluated with a Shore D durometer (model 673-D, Instruments J. Bot S.A., Barcelona, Spain) following ISO 868:2003 [29], using fifteen specimens per formulation. Per the established protocol, standard deviations and one-way analysis of variance (ANOVA) were reported for each mechanical property. Toughness was obtained from the area under the tensile stress–strain curves.

### 2.5. Chemical Characterization

Chemical characterization of the blends was performed by attenuated total reflectance Fourier-transform infrared spectroscopy (FTIR-ATR) to assess interactions between TPS and PVA. Spectra were recorded on a PerkinElmer Spectrum BX FTIR spectrometer equipped with a Pike MIRacle ATR accessory (Beaconsfield, UK). All blends were analyzed in the range 4000–450 cm^−1^ with a resolution of 16 cm^−1^ and 20 scans.

### 2.6. Surface Characterization

Surface morphology was examined by scanning electron microscopy (SEM) on the fractured surface of each formulation. Fracture surfaces were observed using a Phenom SEM (FEI, Eindhoven, The Netherlands) operated at 5 kV. Prior to imaging, samples were sputter-coated with a gold–palladium layer using an Emitech SC7620 coater (Quorum Technologies, East Sussex, UK).

### 2.7. Disintegration Under Composting Conditions

Disintegration under composting conditions was assessed according to ISO 20200:2016 [30] over a thermophilic period of 110 days. The dry solid residue consisted of 10 wt% commercial compost (Mantillo, Spain), 30 wt% rabbit food, 10 wt% starch, 5 wt% sugar, 1 wt% urea, 4 wt% corn oil, and 40 wt% sawdust, adjusted to 55 wt% water content and placed in plastic containers. TPS/PVA blends were compression-molded at 190 °C into 25 cm^2^ films with an average thickness of 2 mm and buried at a depth of 5 cm in the moist solid residue. The reactors were incubated under aerobic conditions at 58 ± 2 °C in an air-circulating oven. The compost was periodically mixed to ensure oxygen supply, and water was added as needed to maintain the prescribed humidity. Specimens were removed at selected times, gently rinsed with distilled water, dried at 40 °C for 48 h, and weighed. Visual changes were recorded for all recovered samples.

Disintegration kinetics were modeled using a modified Avrami equation (Equation (2)):

(2) $$X\left(t\right)=1-\exp\left(-kt^{n}\right),$$

where X(t) is the fractional disintegration at time t (days), k is the rate constant that quantify the speed of disintegration, and n is the Avrami exponent that is related to the disintegration mechanism.

Kinetic fitting was applied only to blends containing ≥30 wt% TPS, for which sufficient data points were available. For PVA-rich compositions (e.g., T20/P80, T10/P90, T0/P100), which disintegrated almost completely within the first 1–2 days, the limited early-time data did not allow reliable parameter estimation; thus, kinetic analysis focused on formulations exhibiting more gradual disintegration governed by combined dissolution, hydrolysis, and microbial erosion.

## 3. Results

### 3.1. Thermal Behavior of the TPS/PVA Blends

**Figure 1:**
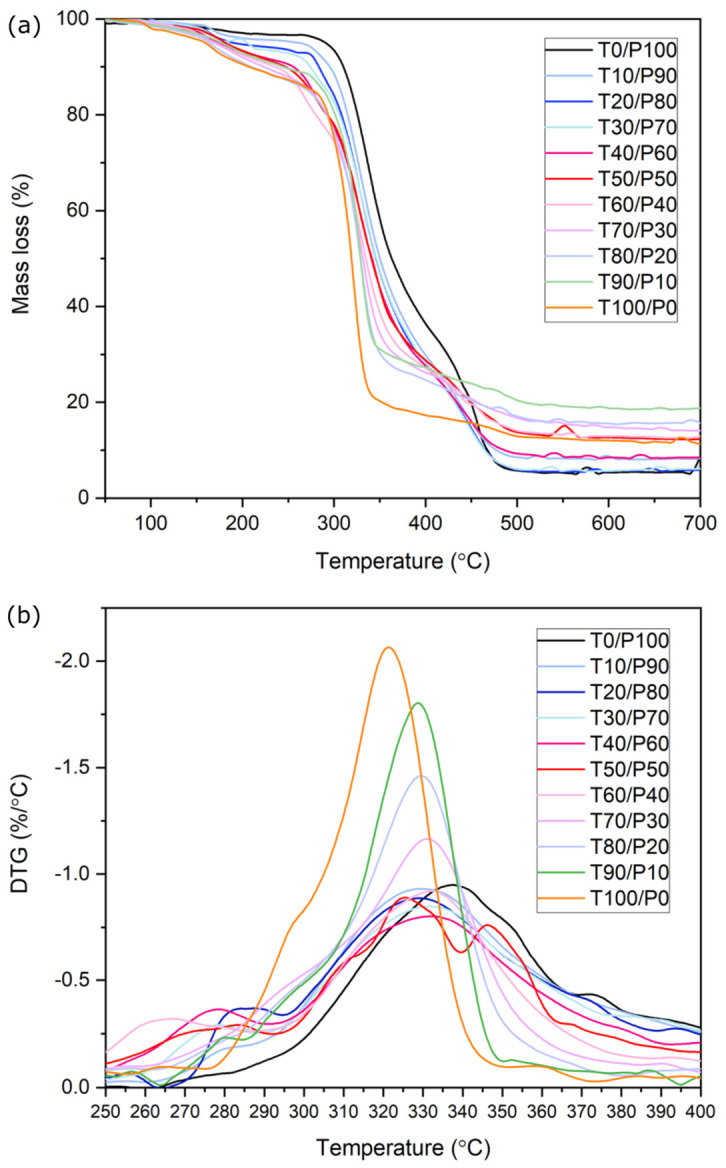
Thermogravimetric analysis of TPS/PVA blends: (**a**) TGA and (**b**) DTG curves. TGA and DTG curves were smoothed using a 50-point Savitzky–Golay method in OriginPro^®^ 2015.

Thermogravimetric analysis (TGA) curves (Figure 1a) reveal the different stages of thermal degradation for neat TPS, neat PVA, and their blends. Neat TPS and TPS-based samples show an initial mass loss around 100 °C, associated with moisture removal. Between 250 °C and 400 °C, another degradation stage occurs, corresponding to the decomposition of –OH groups. This temperature range can be ascribed to the first degradation stage of neat PVA specimens, which is also due to –OH group decomposition, in agreement with previous reports [31,32]. Neat PVA and the T10/P90 blend exhibit the highest onset degradation temperatures (T_5%_) of 290.5 °C and 262 °C, respectively. Increasing TPS content in the blends lowers these temperatures, indicating that TPS is primarily responsible for the reduction in thermal stability, consistent with the lower T_5%_ observed for neat TPS. The reduction in thermal stability observed in TPS-rich blends has practical implications for their processing window and end-use suitability: these formulations are most appropriate for ambient-temperature applications such as flexible packaging or agricultural films, where elevated thermal exposure is not anticipated [5].

Derivative TGA (DTG) curves (Figure 1b) provide further insight into the degradation mechanisms of the blends. T_max_ values for all blends lie between those of the neat components, as expected for binary systems. Blends with higher TPS contents display prominent DTG peaks at lower temperatures (300–330 °C), consistent with the primary degradation of starch. As TPS content decreases and PVA content increases, these peaks shift toward higher temperatures and decrease in intensity, reflecting enhanced thermal stability driven by the intrinsic resistance of PVA to thermal degradation. Similar trends have been reported for PVA/starch-based materials prepared by water-assisted methods, where starch-containing formulations degrade at lower temperatures [33].

Differential scanning calorimetry (DSC) results are summarized in Table 2. For PVA-rich blends (T30/P70 to neat PVA), T_cPVA_ ranges from 129.9 to 133.3 °C, whereas increasing TPS content causes T_cPVA_ to decrease to 118.2 °C for T60/P40. In parallel, ΔH_cPVA_ increases with PVA content, indicating more efficient crystallization in PVA-rich formulations. These trends suggest that high TPS contents hinder PVA crystallization, likely through steric effects or partial phase separation that restrict PVA chain mobility. The second heating scan was used to determine T_gPVA_, T_mTPS_, T_mPVA_, and ΔH_mPVA_, and to calculate Xc_PVA_ via Equation (1); in some blends, these parameters could not be reliably obtained due to low polymer content or poorly defined transitions.

These thermal results are consistent with published data for TPS/PVA systems. The onset degradation temperature of neat PVA (290.5 °C) [34], and the two-stage degradation profile [35]—moisture loss below 100 °C followed by –OH group decomposition between 250–400 °C—are in agreement with values reported for solvent-cast PVA/TPS films of comparable composition, where degradation occurs in the 306–332 °C range and TPS decomposition appears as a shoulder on the main PVA DTG peak [36]. Furthermore, the reduction in thermal stability with increasing TPS content, reflected in the decreasing T_5%_ values (Table 2), is consistent with findings from recent reviews on PVA film thermal stability, where blending with natural polymers consistently lowers the onset degradation temperature relative to neat PVA [35]. The DSC crystallization temperatures (T_cPVA_ ~118–133 °C) are also comparable to values reported for melt-extruded TPS/PVA composites, where T_c_ ~97–129 °C was observed depending on composition and plasticizer content [14].

The lowest T_gPVA_ (59.4 °C) was measured for neat PVA, probably due to partial plasticization by residual moisture or additives, and is lower than the commonly reported T_g_ of ≈69 °C for pure PVA. With increasing TPS content, T_gPVA_ decreases and approaches a plateau around 20 °C for blends containing ≥60 wt% TPS. This reduction indicates a plasticizing effect of TPS on PVA, associated with increased free volume and chain mobility, and is consistent with limited intermolecular compatibility in which TPS partially plasticizes PVA without full miscibility. Additionally, it should be considered that part of this T_g_ depression may arise from partial glycerol migration from the TPS phase into the PVA-rich domains during melt processing. Given the strong thermodynamic affinity between glycerol’s hydroxyl groups and PVA’s hydroxyl-rich backbone, glycerol redistribution is plausible under the melt-blending conditions employed here, and has been reported to reduce PVA crystallinity and promote phase separation at higher glycerol loadings in starch/PVA systems [15,16]. Both effects—intrinsic TPS–PVA compatibility and potential glycerol redistribution—may therefore contribute jointly to the observed reduction in T_gPVA_ and X_cPVA_ in TPS-rich formulations. T_mTPS_ was determined for blends containing ≥40 wt% TPS and varied only slightly between 107.1 and 107.5 °C, showing that TPS melting behavior is largely insensitive to composition.

T_mTPS_ was determined for blends containing ≥40 wt% TPS. In blends with higher PVA contents, evaluation of T_mTPS_ was impeded by the more amorphous character of TPS and overlap with PVA transitions. Where measurable, T_mTPS_ varied only slightly between 106.9 and 107.5 °C, indicating that TPS melting behavior is largely insensitive to composition. Despite the high concentration of TPS in TPS-rich blends, the slight shift in the TPS melting region with increasing PVA content is noteworthy, as it reflects partial interfacial interaction between the two phases rather than purely independent thermal behavior [37], consistent with the FTIR and SEM results.

**Figure 2:**
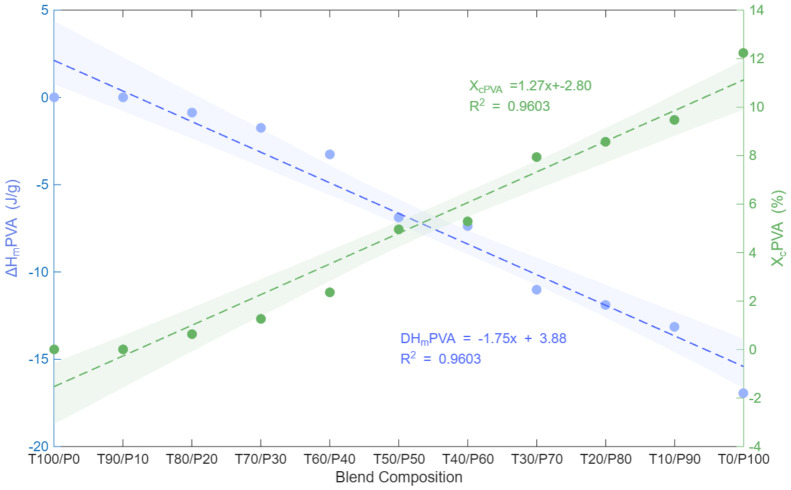
Melting enthalpy (ΔHm) and degree of crystallinity (Xc) of the PVA phase in TPS/PVA blends as a function of composition, determined by differential scanning calorimetry (DSC).

The melting temperature of neat PVA (176.6 °C) is slightly higher than the literature value of ≈165 °C, likely due to batch variability or residual crystallinity. As TPS content increases, T_mPVA_ decreases, reaching a minimum of 150.9 °C for T80/P20. For blends from T0/P100 to T30/P70, a single melting peak is observed in the PVA region, indicating that TPS does not form a separate crystalline phase detectable by DSC in these compositions. By contrast, blends from T40/P60 to T80/P20 display multiple melting endotherms, suggesting partial phase separation or distinct PVA crystalline domains influenced by the TPS phase, consistent with reduced compatibility and a more heterogeneous microstructure. The degree of crystallinity X_cPVA_ increases from 0.63% in T80/P20 to 12.24% in neat PVA (Figure 2), reflecting enhanced chain ordering at higher PVA contents. This behavior contrasts with reports where small amounts of starch or TPS act as nucleating agents and increase crystallinity in other matrices, such as poly(lactic acid). In the present blends, the comparatively high TPS contents dominate morphology and thermal response, suppressing the crystallization capacity of PVA [38].

The nearly linear increase in X_cPVA_ and the corresponding linear decrease in ΔH_mPVA_ with PVA content (Figure 2) indicate an essentially additive, non-synergistic contribution of PVA to crystallinity, consistent with independent crystallization of the PVA phase and the absence of significant nucleating or anti-nucleating effects from TPS. From a thermodynamic perspective, this additive behavior suggests that the Flory–Huggins interaction parameter (χ) between TPS and PVA is close to zero or slightly positive, implying a weakly interacting system with limited thermodynamic driving force for either full miscibility or macroscopic phase separation [39,40]. In a fully miscible blend, one would expect either enhanced nucleation or strong crystallinity suppression beyond the linear trend [41]; the strictly proportional response observed here instead points to largely independent crystallization of the PVA phase, consistent with the partial compatibility established by FTIR and SEM [42].

### 3.2. Mechanical Performance of the TPS/PVA Blends

**Figure 3:**
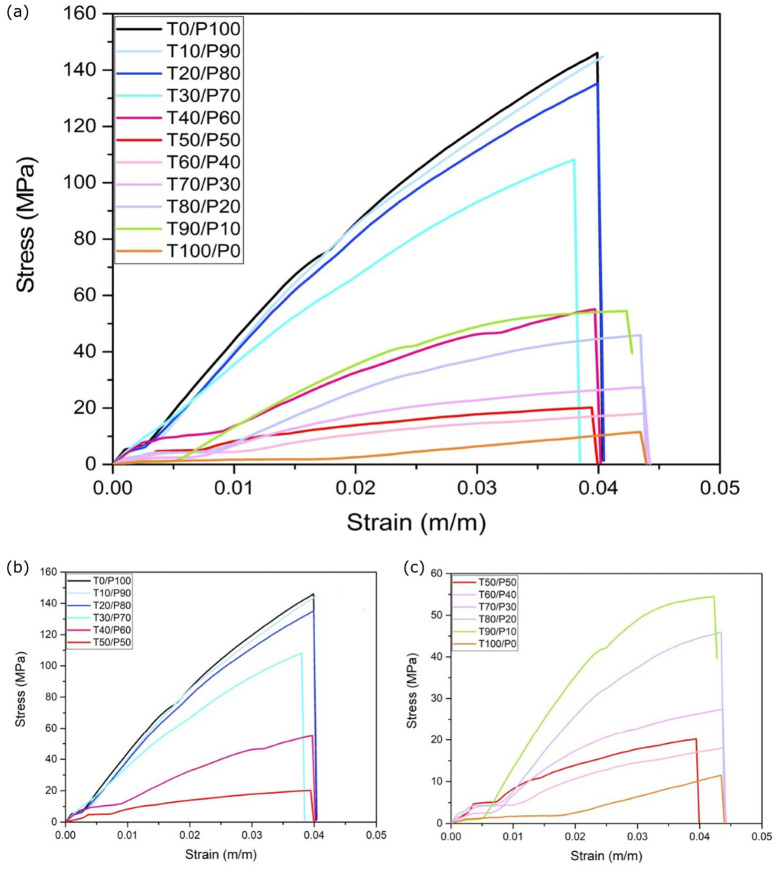
Tensile stress–strain curves of TPS/PVA blends: (**a**) full compositional range, (**b**) PVA-rich formulations (≥50 wt% PVA), and (**c**) TPS-rich formulations (>50 wt% TPS).

Figure 3 presents the tensile stress–strain curves of the blends. The graphic shows that the mechanical response of TPS/PVA blends splits into two regimes. TPS-rich blends (≤50 wt% PVA) are stiff and relatively brittle, whereas PVA-rich blends (>50–60 wt% PVA) become progressively stronger and more ductile.

Formulations with predominant TPS content exhibited the highest stiffness and tensile strength. However, low PVA additions (T90/P10, T80/P20) produced only minor strength increases, indicating limited reinforcement efficiency at these concentrations and thus higher material cost without significant mechanical benefit. In contrast, PVA-rich blends (>70 wt%) show pronounced increases in tensile strength and Young’s modulus, consistent with enhanced interfacial adhesion and partial phase compatibility between TPS and PVA, in agreement with the DSC results.

As with tensile strength, Young’s modulus remained nearly constant for TPS-rich blends, whereas it increases markedly once PVA becomes the dominant phase, which is consistent with improved phase compatibility. The reduction in mechanical strength below 60 wt% PVA can be attributed to the higher flexibility introduced by TPS, similar to the modulus decrease reported when TPS is used to modify polylactic acid [43]. In the present blends, elongation at break changes less than modulus, but the progressive decrease in Young’s modulus with increasing TPS content is clear.

**Figure 4:**
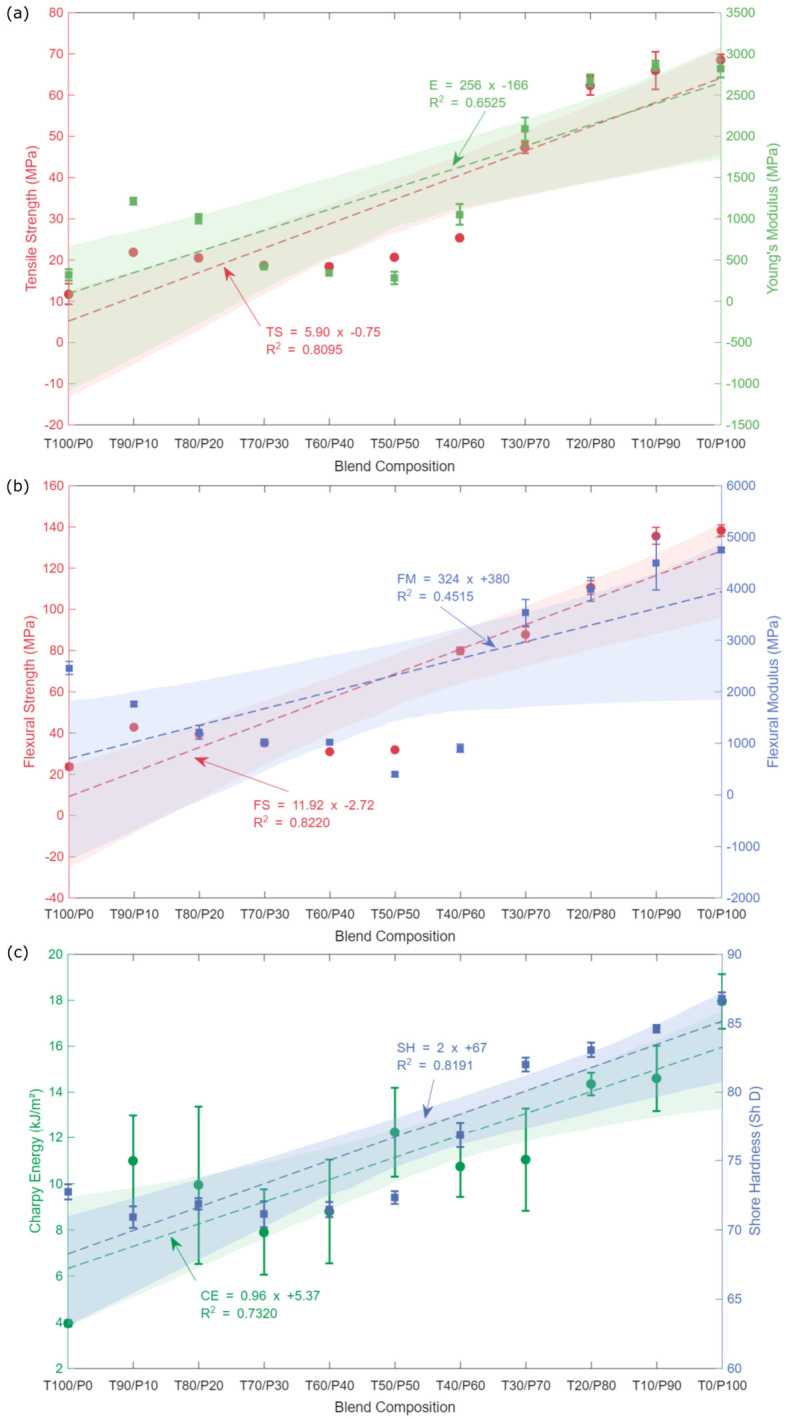
Composition-dependent mechanical properties of TPS/PVA blends: (**a**) tensile strength, Young’s modulus, and elongation at break; (**b**) flexural strength and flexural modulus; and (**c**) impact strength and Shore D hardness. Lines represent linear regressions used as descriptive tools to visualize global composition dependence. In (**a**), red circles correspond to tensile strength (left axis) and green squares to Young’s modulus (right axis); in (**b**), red circles represent flexural strength and blue squares flexural modulus; in (**c**), green circles represent Charpy impact strength and blue ssquares Shore D hardness.

Figure 4 summarizes these trends quantitatively. In TPS-rich formulations, increasing PVA content decreases tensile strength and modulus to a minimum at T50/P50, while elongation at break increases. Above ~60 wt% PVA, this behavior reverses: tensile strength and modulus increase with PVA content, and elongation at break decreases. The linear regressions shown in Figure 4 are used only as descriptive tools: they quantify the overall slope of each property across the full composition range and facilitate comparison between tensile strength, modulus, elongation at break, and hardness. They are not intended as mechanistic models but as a compact way to visualize the global composition dependence on the mechanical response.

From a practical standpoint, the reduction in tensile strength observed in TPS-rich formulations constitutes a meaningful limitation for packaging applications, where mechanical integrity under load, handling, and transport conditions is required. Consequently, TPS-rich blends are better suited for low-demand applications such as agricultural mulch films or single-use disposable items, where end-of-life biodisintegration is the primary design criterion and mechanical demands are minimal. For packaging applications, PVA-rich formulations (≥60 wt% PVA) represent the more appropriate choice within this blend system, offering the necessary combination of tensile strength, stiffness, and ductility.

Toughness, obtained from the tensile curves, also depends on composition. T90/P10 and T80/P20 blends show enhanced toughness, consistent with favorable compatibility at low PVA loadings, while toughness decreases up to T40/P60 (two crystalline phases), then increases again above T30/P70, suggesting improved compatibility in PVA-dominated blends.

Flexural strength and modulus follow similar patterns, reaching maximum values for PVA-rich compositions (≥T30/P70). Increasing PVA content in TPS-rich formulations does not significantly improve flexural strength and even reduces it up to T50/P50, whereas both parameters increase once PVA is the majority phase, in agreement with the tensile and DSC results [24]. Impact strength decreases with increasing TPS content; neat PVA (T0/P100) shows the highest impact resistance, and most differences between intermediate blends are not statistically significant [44]. Finally, Shore D hardness increases with PVA content and is highest for PVA-rich blends (≥70 wt%), mirroring the increase in PVA crystallinity, while TPS addition reduces hardness, as reported for other TPS-based blends [19,45].

These mechanical values are in reasonable agreement with data reported for comparable TPS/PVA systems. Solvent-cast PVA/TPS films at a 1:1 ratio have shown tensile strength values up to 37.2 MPa and elongation at break of ~197% [36], which are higher than those of our injection-molded T50/P50 blend; this difference is attributable to the artificial compatibility enhancement inherent to solvent-based processing, consistent with the methodological gap identified in the Introduction. For melt-processed TPS/PVA blends—the processing route most comparable to the present work—tensile and toughness properties competitive with commercial LDPE have been recently reported, with mechanical performance retained across 10 reprocessing cycles, outperforming PLA and PHB under equivalent conditions [1]. The tensile strength of TPS-rich blends (~18 MPa) is consistent with values reported for PVA-reinforced TPS systems, where tensile strength increased from 2.13 MPa for neat TPS to 20.98 MPa upon PVA fiber incorporation [46]. The composition-dependent elongation trends observed here are also supported by PVA/tapioca TPS bioplastic film studies developed for food packaging applications [32]. Together, these comparisons support the reliability and practical relevance of the mechanical trends found in this work.

### 3.3. Chemical Characteristics of the TPS/PVA Blends

**Figure 5:**
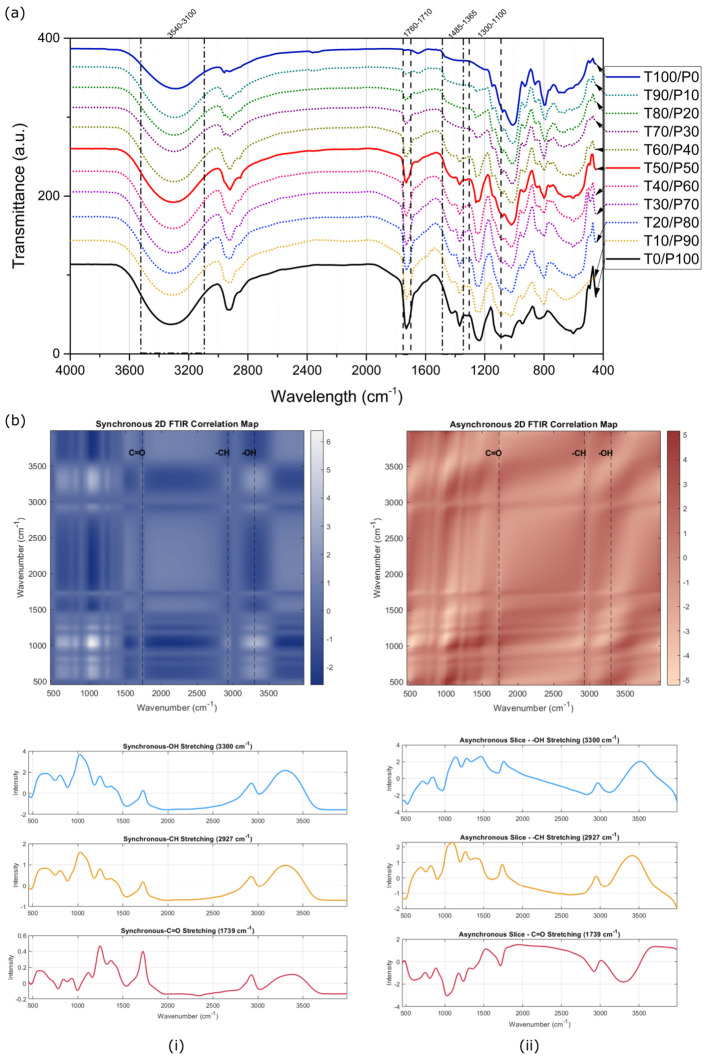
Infrared spectroscopic characterization of TPS/PVA blends: (**a**) attenuated total reflectance Fourier−transform infrared (ATR−FTIR) spectra of neat TPS, neat PVA, and their blends, with representative absorption bands indicated; (**b**) two-dimensional correlation spectroscopy (2D−COS) maps with intensity slices extracted at 3300 cm^−1^ (O–H stretching), 2927 cm^−1^ (C–H stretching), and 1739 cm^−1^ (C=O stretching): (**i**) synchronous and (**ii**) asynchronous maps.

Fourier-transform infrared (FTIR) spectroscopy was used to identify functional groups and assess molecular interactions in the blends. Figure 5 shows the spectra in the 4000–400 cm^−1^ range. Neat TPS, neat PVA, and their blends exhibit broad –OH stretching bands between 3540 and 3100 cm^−1^, consistent with previous reports [32,47]. Although 1D FTIR spectra alone does not allow a definitive assessment of blend compatibility, combined analysis of thermal, mechanical, and morphological data indicates partial compatibility between the two phases, without evidence of strong specific interactions (this concept is discussed in detail at the end of this section).

To obtain more detailed information from the same FTIR data set, we applied two-dimensional correlation spectroscopy (2D-COS) using blend composition as the perturbation variable. In 2D-COS, synchronous maps highlight bands that change in intensity together as composition varies, whereas asynchronous maps highlight bands that change sequentially (i.e., one responds earlier or more strongly than another as composition is changed). In the synchronous maps (Figure 5a), positive off-diagonal cross-peaks between the O–H stretching region (~3300 cm^−1^) and the C=O and C–O regions (~1700 and ~1100 cm^−1^) indicate that hydroxyl and carbonyl groups respond cooperatively to changes in TPS/PVA ratio, consistent with composition-dependent hydrogen-bonding interactions. The corresponding asynchronous maps (Figure 5b) show weaker, but non-zero, cross-peaks between these regions, suggesting that the hydrogen-bonding environment of TPS –OH groups is perturbed first, followed by a more gradual reorganization or partial solubilization of PVA domains. Taken together, these features indicate that TPS and PVA interact through relatively weak, localized hydrogen bonding that is sensitive to composition, but they do not form a strongly coupled, fully miscible network.

While the 2D-COS analysis indicates that the hydrogen bonding environment evolves with composition, the absence of pronounced peak shifts in the 1D spectra confirms that these interactions remain moderate and localized rather than indicative of strong molecular-level bonding. When this FTIR evidence is combined with the observation of a single, composition-dependent T_g_ for PVA in DSC and the morphologically homogeneous, phase-separated-free micrographs in SEM, a coherent picture of partial compatibility emerges: the blends are sufficiently compatible at the microscale to form continuous, defect-free materials, but do not exhibit full miscibility at the molecular scale. This level of interaction is enough to ensure stable processing and morphological integrity in film and part fabrication, while the absence of strong interfacial coupling explains the gradual, composition-driven changes in mechanical and disintegration behavior rather than pronounced synergistic effects.

### 3.4. Microstructural Morphology TPS/PVA Blends

**Figure 6:**
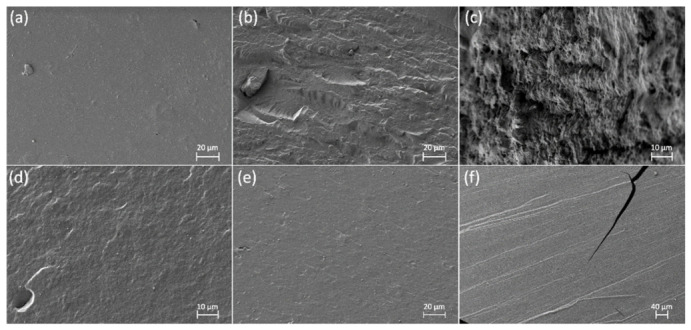
Scanning electron microscopy (SEM) images of the cryo-fractured surface (**a**) T100/P0 (neat TPS), (**b**) T80/P20, (**c**) T70/P30, (**d**) T60/P40, (**e**) T30/P70, and (**f**) T0/P100 (neat PVA) blends.

SEM micrographs (Figure 6) show morphologically homogeneous structures for all blends, with no evidence of macroscopic phase separation. The description below focuses on TPS-rich compositions to highlight the main changes. Neat TPS and neat PVA exhibit uniform fracture surfaces, as expected, with neat PVA displaying a more rigid, cracked morphology than TPS (Figure 6a,f). All blends retain a generally homogeneous morphology without distinct phase domains. For example, T30/P70 shows a brittle fracture pattern similar to neat PVA (Figure 6e), whereas blends between T60/P40 and T80/P20 (Figure 6b–d) present smoother fracture surfaces, indicative of improved interfacial adhesion and consistent with the observed morphological homogeneity.

### 3.5. Composting of TPS/PVA Blends

**Figure 7:**
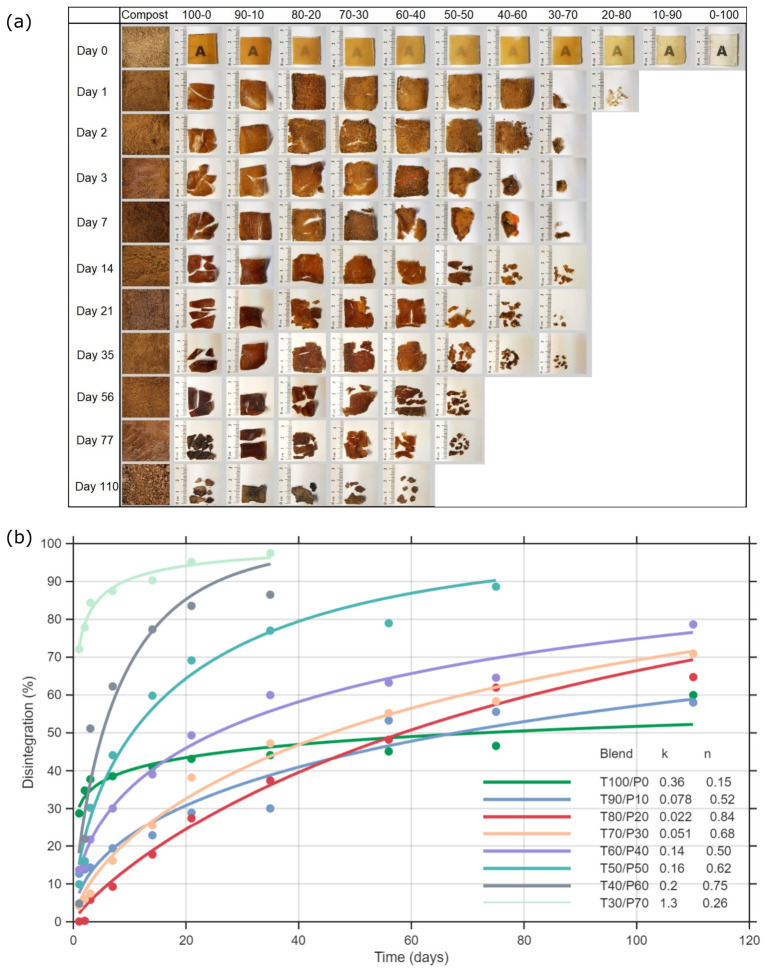
Disintegration behavior of TPS/PVA blends under simulated composting conditions (ISO 20200:2016): (**a**) photographic evolution of representative samples recovered at selected incubation times; (**b**) disintegration degree as a function of incubation time, with Avrami model fits shown for compositions containing ≥30 wt% TPS.

Figure 7 shows the visual evolution of TPS/PVA samples recovered at different incubation times. PVA-rich blends disintegrated almost completely within two days, in agreement with ISO 20200:2016 [30], which considers dissolution a valid form of disintegration when no visible residue remains. In contrast, increasing TPS content led to more gradual disintegration. Formulations T30/P70 and T40/P60 reached mass losses of 97% and 86%, respectively, after 35 days, suggesting a process governed by both dissolution and microbial activity. The T50/P50 blend lost ≈88% of its mass after 75 days, indicating slower disintegration at higher TPS contents. Blends with predominantly TPS, including neat TPS, were monitored for 110 days and showed final disintegration between 58% and 78%. In all cases, the fastest mass loss occurred during the first 14 days, followed by a slower stage, a kinetic behavior consistent with previous reports where PVA/starch composites show rapid initial disintegration followed by a stabilization phase [48].

Overall, all formulations showed substantial disintegration under the conditions specified in ISO 20200:2016 [30]. A critical interpretation is nevertheless required. The rapid mass loss of PVA-rich blends arises mainly from their high-water solubility and consequent dissolution. This process satisfies the physical disintegration criterion of ISO 20200 but does not, by itself, demonstrate microbial biodegradation. Thus, although all blends disintegrate effectively, additional tests in accordance with standards such as EN 13432:2000 [49] would be needed to assess full compostability and quantify the relative contribution of true biodegradation at each stage.

The Avrami model was applied only to blends containing ≥30 wt% TPS, for which sufficient data points were available, and the model is physically appropriate. It was not applied to PVA-rich formulations (T20P80, T10P90, T0P100) because their near-instantaneous dissolution within 1–2 days yielded insufficient data for reliable parameter fitting and do not conform to the nucleation-and-growth or diffusion-controlled mechanisms the model describes; kinetic analysis was therefore not performed for these compositions. Consequently, the kinetic discussion focuses on compositions that exhibit more gradual disintegration, governed by a combination of dissolution, hydrolysis, and microbial erosion.

Fitted parameters revealed clear composition-dependent trends. In PVA-rich blends, low Avrami exponents (*n* < 1) and high rate constants k indicates surface-controlled dissolution processes, consistent with the high water solubility of PVA. SEM images (Figure 6e,f) and visual observations (Figure 7) support this interpretation by showing morphologies that facilitate rapid water ingress, while FTIR results (Figure 5) indicate limited hydrogen bonding, further favoring physical disintegration. With increasing TPS content, n increases and k decreases, consistent with diffusion- or erosion-controlled disintegration. TPS-rich blends exhibit greater structural cohesion and lower porosity, together with stronger intermolecular interactions inferred from FTIR, leading to slower, multi-step disintegration involving bulk hydrolysis and microbial erosion over time. In summary, the Avrami model captures the transition from rapid dissolution to slower, diffusion-controlled disintegration across the compositional range.

## 4. Conclusions

TPS/PVA blends were successfully produced via solvent-free extrusion and injection molding, yielding morphologically homogeneous materials with partial compatibility across the full compositional range. The processing route provided uniform, defect-free samples under conditions directly translatable to industrial scale-up. Thermal and mechanical analyses revealed clear composition-dependent transitions from stiff, TPS-rich to ductile, PVA-rich behavior, defining a tunable window in which stiffness, toughness, and ductility can be balanced. DSC and FTIR results confirmed partial compatibility with limited hydrogen bonding, while SEM observations corroborated morphological homogeneity and supported the structure–performance relationships inferred from mechanical data. All compositions exhibited substantial disintegration under composting conditions; however, in PVA-rich blends this process was largely driven by water solubility and dissolution rather than microbial degradation.

Taken together, these findings provide a comprehensive framework linking composition, processing, structure, performance, and disintegration behavior in solvent-free TPS/PVA systems. They indicate that these blends are promising candidates in contexts where rapid physical disintegration is desired, while the limitations associated with dissolution-driven mass loss and the reduced mechanical performance of TPS-rich formulations must be considered when defining target applications. Specifically, TPS-rich blends are better suited for low-demand applications such as agricultural mulch films or single-use disposable items, whereas PVA-rich formulations (≥60 wt% PVA) are more appropriate for packaging applications requiring adequate tensile strength and stiffness. Future work should focus on evaluating barrier performance (WVTR and OTR, following ISO 15106-3:2003 [50] and ISO 15105-1:2007 [51]) and moisture sorption to assess packaging suitability, as well as conducting standardized biodegradation tests based on CO_2_ evolution (ISO 14855-1:2012 [52]) combined with disintegration and ecotoxicity assays according to EN 13432:2000 [49] and ISO 17088:2021 [53], to fully assess compostability compliance and distinguish true biodegradation from dissolution-driven mass loss.

## Figures and Tables

**Table 1:** Formulation codes and composition ratios of TPS/PVA blend studied (*n* = 11).

Blend	TPS Content (wt.%)	PVA Content (wt.%)
T100/P0 (neat TPS)	100	0
T90/P10	90	10
T80/P20	80	20
T70/P30	70	30
T60/P40	60	40
T50/P50	50	50
T40/P60	40	60
T30/P70	30	70
T20/P80	20	80
T10/P90	10	90
T0/P100 (neat PVA)	0	100

**Table 2:** Thermal parameters of the TPS/PVA blends determined from TGA and DSC measurements.

TPS/PVA Blends	T_5%_ (°C)	T_max_ (°C)	T_gPVA_ (°C)	T_cPVA_ (°C)	T_mTPS_ (°C)	T_mPVA_ (°C)
T100/P0 (neat TPS)	160.5	322.5	n.d. *	n.d. *	107.5	n.d. *
T90/P10	167.5	327.5	20.6	n.d. *	107.4	n.d. *
T80/P20	165.0	329.0	20.3	n.d. *	107.4	150.9
T70/P30	170.0	331.0	19.3	n.d. *	107.3	151.8
T60/P40	168.5	334.5	31.8	118.2	107.2	161.6
T50/P50	171.5	331.5	37.6	121.9	107.1	162.6
T40/P60	176.0	334.0	38.3	127.9	107.3	166.8
T30/P70	186.0	333.0	48.6	133.3	n.d. *	173.6
T20/P80	190.5	331.5	49.2	133.3	n.d. *	173.5
T10/P90	262.0	333.0	53.8	133.3	n.d. *	175.5
T0/P100 (neat PVA)	290.5	339.0	59.4	129.9	n.d. *	176.6

* Not determined due to insufficient content of the corresponding component for reliable signal detection.

## Data Availability

The raw data supporting the conclusions of this article will be made available by the authors on request.
